# Interprofessional Education: A Model for Academic and Community Collaboration for Professional Education

**DOI:** 10.1093/geroni/igaa057.012

**Published:** 2020-12-16

**Authors:** Elaine Jurkowski

**Affiliations:** Southern Illinois University at Carbondale, Saint Charles, Missouri, United States

## Abstract

Teamwork and collaboration across disciplines is becoming critically important as we meet the health and human service needs of people growing older and their families. The myriad of competencies, language and tasks specific to each discipline are not easily or intuitively mastered within discipline specific curricula. This presentation aims to provide a model that addresses the curricular needs for course preparation through inter-professional educational strategies. While the traditional IPE components are address, this model also integrates in the training process, education through the lens of the social determinants of health, community collaboration through health and human service networks, population health and public policy. This presentation will lay out the model and articulate specific educational strategies to address each of the dimensions of the model, to include the flip classroom, experiential activities, assessment and intervention tools, panel discussions with community and agency partners and epidemiologic/population health data. This model identifies a unique approach to teaching students and professionals about collaboration across disciplines for the benefits of addressing the needs of an older adult target group. This model also moves the process of teaching interprofessional collaboration and education beyond understanding values and ethics, roles and responsibilities, team care and communication.

